# The effects of weight and physical activity change over 20 years on later-life objective and self-reported disability

**DOI:** 10.1093/ije/dyu013

**Published:** 2014-02-20

**Authors:** Emily D Williams, Sophie V Eastwood, Therese Tillin, Alun D Hughes, Nishi Chaturvedi

**Affiliations:** International Centre for Circulatory Health, Imperial College London, London, UK

**Keywords:** Weight change, Disability, Obesity, Physical activity, Older age

## Abstract

**Background:** Weight and health behaviours are known to affect physical disability; however the evidence exploring the impact of changes to these lifestyle factors over the life course on disability is inconsistent. We aimed to explore the roles of weight and activity change between mid and later life on physical disability.

**Methods:** Baseline and 20-year clinical follow-up data were collected from1418 men and women, aged 58–88 years at follow-up, as part of a population-based observational study based in north-west London. At clinic, behavioural data were collected by questionnaire and anthropometry measured. Disability was assessed using a performance-based locomotor function test and self-reported questionnaires on functional limitation and basic activities of daily living (ADLs).

**Results:** At follow-up, 39% experienced a locomotor dysfunction, 24% a functional limitation and 17% an impairment of ADLs. Weight gain of 10–20% or >20% of baseline, but not weight loss, were associated with increased odds of a functional limitation [odds ratio (OR) 1.69, 95% confidence interval (CI) 1.14-2.49 and OR 2.74, 1.55-4.83, respectively], after full adjustment for covariates. The same patterns were seen for the other disability outcomes. Increased physical activity reduced, and decreased physical activity enhanced the likelihood of disability, independent of baseline behaviours and adiposity. The adverse effects of weight gain appeared to be lessened in the presence of increased later-life physical activity.

**Conclusion:** Weight and activity changes between mid and later life have strong implications for physical functioning in older groups. These findings reinforce the importance of the maintenance of healthy weight and behaviour throughout the life course, and the need to promote healthy lifestyles across population groups.

**Key Messages**

- At least 17% of a multi-ethnic later-life population experienced some level of objective or subjective disability.
- Weight gain from mid to later life is associated with greater objective and subjective disability, independently of baseline weight, physical activity, sociodemographic factors and morbidity.
- Greater physical activity in later life is protective for disability, regardless of weight or physical activity levels in mid life.

## Introduction

Obesity prevalence continues to rise in most countries in the world, with the number of obese people projected to reach 573 million worldwide by 2030.^1^ With an ageing global population, this is likely to adversely impact on physical disability.^2^^,^^3^

As well as absolute weight, weight change predicts physical disability, in particular amongst older adults.^4–7^ The evidence, however, is inconsistent, with some studies showing a stronger relationship between weight loss and disability than weight gain^4^^,^^7^^,^^8^and vice versa.^9^^,^^10^ The majority of the existing studies include self-reported weight from at least one data time point^4^^,^^5^^,^^9–12^ and only subjective measures of disability.^4^^,^^5^^,^^7–9^ One of the few studies to include objective measures of weight change and disability showed that weight loss and gain greater than 5% of baseline values were related to walking limitations.^13^

In addition to weight, health behaviours such as physical inactivity and smoking play an important role in the development of physical disability.^14^ The few existing studies exploring behaviour over time and risk of disability have shown conflicting findings; sustained physical activity over decades was shown to have a protective effect on disability in one study^15^ but not in another.^16^ In addition, there may be a biological interaction between the effects of weight and physical activity change on disability, with weight increase in the presence of enhanced physical activity being due to accrual of lean, rather than fat mass.^17^

We hypothesized that weight gain and/or weight loss between mid and later life would be associated with the development of physical disability. In addition, it was hypothesized that reduced physical activity over the 20-year follow-up would have an effect on disability independent of weight change.

## Methods

### Participants

The Southall and Brent REvisited (SABRE) study is a tri-ethnic (European, South Asian and African Caribbean) community-based prospective study recruited from primary care registers in north-west London between 1988 and 1991.^18^ Local research ethics committees [baseline: Ealing, Hounslow and Spelthorne, and University College London research ethics committees; follow-up: St Mary’s Hospital Research Ethics Committee (ref.07/H0712/109)] approved the study. Written informed consent was provided by all participants. At baseline, participants were aged between 40 and 69 years, and included 4857 people of European (*n* = 2346), South Asian (*n* = 1710) and African Caribbean (*n* = 801) ethnic origin. Ethnicity was identified on the basis of parental ancestry. Traced survivors were invited to take part in the follow-up study (2008–11), 20 years after the baseline survey, when participants were aged between 58 and 88 years.

### Baseline measurements

Weight and height were measured with the participant barefoot, wearing a hospital gown and standing straight with the head level, using Soehnle scales and a stadiometer, respectively.

A self-administered questionnaire included sociodemographic, behavioural and medical history items. Physical activity was measured using total weekly energy expended (MJ) in sport, walking and cycling, using questions and energy expenditure estimates.^19^ Sedentary behaviour was measured by self-reported hours of television viewing per week. Disability was measured by self-report of activity-limiting conditions and dichotomized into presence or absence of limitation. Socio-economic position (SEP) was dichotomized into manual and non-manual occupations, according to the 1980 Registrar General’s classification.^18^ Arthritis and asthma were identified from participant report; hypertension from treatment or clinic reading of ≥140/90 mmHg; diabetes from medication, primary care records or oral glucose tolerance test; coronary heart disease (CHD) from primary care records; and stroke from participant report or primary care records, as previously described.^18^

### Follow-up measurements

Clinic attendees completed a similar questionnaire to baseline, and underwent a series of clinical and anthropometric measurements.^18^ Weight, height and physical activity were measured using procedures identical to those in the baseline assessments. Disability was measured using the objective ‘up and go’ test, as well as self-reported functional limitation and activities of daily living (ADLs) (see Table 1).

**Table 1.** Follow-up assessment of disability

Variables	Scale/measurement	Categorization
Objective disability	Locomotor function—‘Up and Go’ test,^35^ standardized measure of functional leg strength, power and balance. Incorporates basic mobility movements needed for successful ageing	Timed test involved participants getting up from a chair, walking three metres, turning around, walking and sitting back down; the threshold of ≥12 s was used to classify locomotor dysfunction^36^^,^^37^
Subjective disability	Functional limitation	Impairment recorded if participants reported limitation with ≥1 of following: (i) walking unaided without stopping and discomfort; (ii) walking up and down a flight of 12 stairs without resting; (iii) bending down to pick up a shoe from the floor^38^
	Activities of daily living impairment^38^	Impairment recorded if participants reported limitation with ≥1 of following: (i) walking across a room; (ii) getting in and out of bed; (iii) getting in and out of a chair; (iv) dressing and undressing oneself; (v) bathing or showering; (vi) self-feeding; (vii) getting to and using the toilet^38^

### Statistical analyses

Age- and sex-adjusted analyses of covariance and logistic regression were used as appropriate to compare the baseline (1988–91) characteristics of responders with non-responders (traced survivors who did not participate in follow-up). Subsequent analyses included only those people with complete data for the covariates of interest (*n* = 1418, for objective locomotor function analyses *n* = 1393). Baseline characteristics were compared across groups according to self-reported functional limitation status (chosen for comparability with most other studies using functional limitation as the disability outcome^4^^,^^6^^,^^13^^,^^20–23^), using age- and sex-adjusted analyses of covariance, logistic regression and Mann Whitney U-tests as appropriate.

Binary logistic and linear regression analyses explored changes in: (i) weight; (ii) body mass index (BMI) trajectory; (iii) physical activity; and (iv) physical activity trajectory as risk factors for the three separate disability outcomes of objective locomotor dysfunction, self-reported functional limitation and ADL impairment. In the first set of models, the impact of weight change categories (>10% loss, 5–10% loss, 5% loss to 5% gain (i.e. stable = reference category), 5–10% gain, 10–20% gain and >20% gain) was tested with age-, sex- and ethnicity-adjustment, followed by further adjustment for baseline covariates (socio-economic position (SEP), weight, height, physical activity, smoking, sedentary behaviour, self-rated health, CHD, diabetes, hypertension, asthma and arthritis). The impacts of BMI trajectories (reference category = healthy throughout, using standard thresholds of healthy <25 kg/m^2^, overweight 25–29.9 kg/m^2^ and obese ≥30 kg/m^2^),^24^ physical activity change quintiles (reference category = stable), physical activity as a linear term and physical activity trajectories (based on transitions between baseline and follow-up tertiles of physical activity) were examined with similar levels of adjustment. Finally we evaluated the combined impact of both weight and physical activity change on disability. We looked at weight change x physical activity change interaction terms in models of disability outcomes, and examined graphically the proportion of participants with each disability outcome by category of weight and physical activity change. Sex and ethnicity by change variable interaction terms were tested in the models; however no interactions were observed.

A range of sensitivity analyses were conducted to test the robustness of the findings. Firstly, ‘incident’ disability was explored by including only those people free from disability at baseline in the analyses (*n* = 1102); we did not use this strategy in the main analyses as the baseline measure of disability was not validated and did not directly correspond with follow-up measures. Secondly, instead of change variables, the baseline and follow-up weight values were included in the same models as the main analyses. To examine the role of smoking status on weight change (given the common consequence of weight gain following smoking cessation^25^), the models were repeated stratified by smoking status over time. Lastly, analyses were repeated stratified by age within the sample (younger age group 40–49 years, older age group 50–66 years) to see if effects varied with age. All analyses were performed using SPSS version 18.

## Results

### Comparison of baseline characteristics between responders and non-responders at follow-up

Of the 4857 participants at baseline, 1124 had died before follow-up. Of the remainder, 38% attended clinic. The survivors who did not participate in follow-up were older (*P* < 0.001), more likely to be female (*P* = 0.001) or to have worked in a manual occupation (*P* < 0.001) than participants. They were also more likely to have smoked (*P* < 0.001), be of heavier weight (*P* < 0.001) and to have diabetes (*P* = 0.001) and hypertension (*P* = 0.018). There were no group differences in prevalence of CHD (*P* = 0.59), asthma (*P* = 0.21), arthritis (*P* = 0.07) or baseline disability (*P* = 0.73).

### Baseline characteristics of participants in relation to functional limitation status

At follow-up, 39% were found to have objective locomotor dysfunction, 24% a self-reported functional limitation and 17% an impairment of ADLs. Mean weight change between baseline and follow-up was +2.9 kg [standard deviation ((SD) 8.2]. Physical activity was reduced by a mean of 1.7 h per week (SD 7.3). People who developed a self-reported functional limitation during follow-up were older, more likely to be female, to be from an ethnic minority and to have a manual occupation, compared with people with no self-reported functional limitation. They were also more likely to have lower levels of physical activity, be of heavier weight and have greater chronic disease burden (Table 2).

**Table 2.** Baseline characteristics of participants according to follow-up functional limitation status

Characteristics	No functional limitation	Functional limitation	*P*–value*
	(*n* = 1073)	(*n* = 345)	
Age	49.4 ± 6.1	51.5 ± 6.3	<0.001
Male sex (%)	78.0	70.7	0.006
Ethnic group (%)			
White	53.8	29.3	
South Asian	31.2	51.0	<0.001
African Caribbean	15.0	19.7	<0.001
Marital status (%)			
Married/cohabiting	83.9	80.8	0.440
Single	7.6	5.2	
Divorced/separated	6.9	9.0	
Widowed	1.6	4.9	
Manual occupation (%)	56.2	74.5	<0.001
Smoking (%)			
Ex/never	83.5	83.2	0.650
Current	16.5	16.8	
Physical activity (kJ/week)	10.3 (7.0,15.0)	9.5 (4.3,14.1)	<0.001
Sedentary behaviour (h/week)	3.5 ± 1.1	3.4 ± 1.2	0.150
Self-rated health good/very good (%)	74.6	54.2	<0.001
Disability (%)	18.0	34.4	<0.001
Weight (kg)	74.8 ± 12.1	76.6 ± 13.7	0.001
Height (cm)	170.6 ± 8.4	167.0 ± 9.2	<0.001
Body mass index (kg/m^2^)	25.6 ± 3.4	27.5 ± 4.7	<0.001
Diabetes (%)	5.6	14.5	<0.001
Coronary heart disease (%)	2.6	5.5	0.038
Hypertension (%)	19.6	30.1	0.001
Asthma (%)	8.8	11.3	0.270
Arthritis (%)	11.4	23.2	<0.001

Data presented are unadjusted means (SD) unless otherwise stated, with the exception of physical activity which is presented as medians (interquartile range), due to skewed data.

*P-values indicate age- and sex-adjusted group differences.

### Weight change as a risk factor for disability

For all three disability measures, the same patterns were observed, showing a graded increased likelihood of disability with a baseline weight gain of 10–20% and >20% (more marked for self-reported functional limitation and ADL impairment), compared with people who maintained a stable weight throughout mid and later life (Table 3). The majority of these relationships remained after controlling for a range of covariates, including baseline weight, indicating that weight gain was a risk factor for disability independent of mid-life adiposity. Weight loss was not associated with objective or self-reported disability.

**Table 3.** Weight change and cumulative body mass index as risk factors for disability outcomes

Weight change between baseline and follow-up	Objective disability OR (95% CI)	Functional limitation OR (95% CI)	ADL impairment OR (95% CI)
Model 1			
>10% loss (*n* = 103)	1.18 (0.74, 1.89)	1.24 (0.76, 2.02)	0.96 (0.55, 1.69)
5–10% loss (*n* = 156)	0.89 (0.60, 1.32)	0.93 (0.60, 1.44)	0.90 (0.55, 1.48)
5% loss to 5% gain (stable) (*n* = 568)	1	1	1
5–10% gain (*n* = 247)	0.86 (0.61, 1.21)	1.17 (0.80, 1.71)	0.96 (0.56, 1.69)
10–20% gain (*n* = 265)	1.10 (0.79, 1.53)	1.59 (1.10,2.30)**	1.38 (0.91, 2.08)
>20% gain (*n* = 91)	1.68 (1.02, 2.76)*	2.80 (1.64, 4.76)***	1.89 (1.04, 3.43)*
Model 2			
>10% loss (*n* = 103)	0.96 (0.59, 1.56)	0.96 (0.57, 1.61)	0.73 (0.40, 1.31)
5–10% loss (*n* = 156)	0.80 (0.53, 1.20)	0.78 (0.49, 1.25)	0.81 (0.48, 1.36)
5% loss to 5% gain (stable)(*n* = 568)	1	1	1
5–10% gain (*n* = 247)	0.90 (0.64, 1.27)	1.30 (0.87, 1.93)	1.05 (0.67, 1.65)
10–20% gain (*n* = 265)	1.14 (0.81, 1.61)	1.69 (1.14, 2.49)**	1.56 (1.02, 2.41)*
>20% gain (*n* = 91)	1.60 (0.95, 2.69)	2.74 (1.55, 4.83)***	1.85 (1.00, 3.43)*

Category of body mass index (BMI) change between baseline and follow-up			

Model 1			
Stable			
Healthy throughout (*n* = 341)	1	1	1
Overweight throughout (*n* = 392)	1.21 (0.87, 1.68)	1.19 (0.80, 1.78)	1.99 (1.21, 3.25) **
Obese throughout (*n* = 142)	3.22 (2.07, 5.00)***	5.26 (3.26, 8.49)***	5.93 (3.38, 10.40)***
Loss			
Overweight to healthy (*n* = 75)	1.65 (0.94, 2.89)	1.24 (0.67, 2.29)	1.82 (0.87, 3.84)
Gain			
Healthy to overweight (*n* = 226)	1.02 (0.69, 1.51)	1.70 (1.08, 2.67)*	2.37 (1.37, 4.10)**
Overweight to obese (*n* = 197)	2.20 (1.48, 3.28)***	3.68 (2.37, 5.73)***	4.74 (2.80, 8.03)***
Model 2			
Stable			
Healthy throughout (*n* = 341)	1	1	1
Overweight throughout (*n* = 392)	1.15 (0.82, 1.62)	1.10 (0.73, 1.67)	1.91 (1.21, 3.25) **
Obese throughout (*n* = 142)	2.87 (1.80, 4.56)***	4.70 (2.81, 7.87)***	5.93 (3.38, 10.40)***
Loss			
Overweight to healthy (*n* = 75)	1.61 (0.91, 2.85)	1.12 (0.59, 2.14)	1.82 (0.87, 3.84)
Gain			
Healthy to overweight (*n* = 226)	1.01 (0.68, 1.52)	1.73 (1.09, 2.77)*	2.37 (1.35, 4.14)**
Overweight to obese (*n* = 197)	2.05 (1.36, 3.08)***	3.56 (2.25, 5.63)***	4.61 (2.68, 7.91)***

Model 1 included adjustment for age, sex and ethnic group. Model 2 included additional adjustment for baseline smoking, manual occupation, sedentary behaviour, weight (weight change models only), height (weight change models only), physical activity, self-rated health, coronary heart disease, diabetes, hypertension, asthma and arthritis.

*P < 0.05, **P < 0.01, ***P < 0.001. For BMI change analyses, participants in the normal weight to obese category have not been included due to small numbers (*n* = 16).

### Change in BMI as a risk factor for disability

Compared with those people who maintained a healthy BMI throughout mid and later life, the greatest odds of disability were experienced among those who were obese at both baseline and follow-up (Table 3). In addition, moving up a BMI category was generally related to increased disability. There was some evidence that moving from overweight to healthy appeared to increase the odds of disability compared with having a healthy BMI throughout. Patterns of associations between BMI trajectories and disability were similar for each disability measure.

### Physical activity change as a risk factor for disability

There was a graded relationship between physical activity change and disability (Table 4). Age-, sex- and ethnicity-adjusted models showed that a reduction of >6.7 h per week in physical activity between baseline and follow-up was associated with increased odds of all disability outcomes. Participants who reported increases in physical activity of >3.7 h per week had lower odds of self-reported functional limitation. Further adjustment of these findings was not possible due to model instability; however, since the associations between physical activity change and disability appeared to be linear (*P* for trend <0.001 for each outcome), we also looked at models featuring physical activity change as a linear term. In fully adjusted models, there was strong evidence for an inverse relationship between a 1-SD increase in physical activity between baseline and follow-up and all disability outcomes. Using participants in the lowest tertile for physical activity at baseline and follow-up as the referent category, we examined the impact of physical activity trajectories on disability. Those in the highest tertile of physical activity at follow-up had the lowest likelihood of all three disability outcomes in fully-adjusted models, regardless of physical activity tertile at baseline.

**Table 4.** Physical activity change and cumulative physical activity as risk factors for disability outcomes

Physical activity change between baseline and follow-up	Objective disability OR (95% CI)	Functional limitation OR (95% CI)	ADL impairment OR (95% CI)
Model 1 (quintiles)			
Q1: Reduction >6.7 h/wk	1.49(1.01, 2.19)*	2.52 (1.78, 3.59)***	1.82 (1.23, 2.69)**
Q2: Reduction 2.4–6.7 h/wk	1.18 (0.81, 1.73)	1.45 (1.02, 2.07)*	1.23 (0.82, 1.83)
Q3: Stable	1	1	1
Q4: Increase 0.7–3.7 h/wk	1.34 (0.92, 1.97)	1.12 (0.77, 1.61)	1.09 (0.72, 1.63)
Q5: Increase >3.7 h/wk	0.84 (0.57, 1.23)	0.66 (0.45, 0.98)*	0.66 (0.42, 1.03)
Model 1 (linear term)			
1 SD increase	0.85 (0.75, 0.96)**	0.66 (0.59, 0.74)***	0.75 (0.66, 0.84)***
Model 2 (linear term)			
1 SD increase	0.87 (0.77, 0.99)*	0.65 (0.56, 0.76)***	0.72 (0.61, 0.85)***

Trajectories between baseline and follow-up tertiles of physical activity^a^			

Model 1			
Highest baseline, highest follow-up (*n* = 227)	0.23(0.14,0.37)***	0.16(0.10,0.27)***	0.11(0.05,0.22)***
Highest baseline, lowest follow-up (*n* = 146)	1.14(0.67,1.95)	1.63(1.05,2.54)*	1.04(0.66,1.64)
Lowest baseline, highest follow-up (*n* = 114)	0.21(0.11,0.38)***	0.10(0.05,0.21)***	0.12(0.05,0.29)***
Lowest baseline, lowest follow-up (*n* = 290)	1	1	1
Model 2			
Highest baseline, highest follow-up (*n* = 227)	0.25(0.15,0.41)***	0.21(0.11,0.41)***	0.19(0.08,0.42)***
Highest baseline, lowest follow-up (*n* = 146)	1.11(0.62,1.99)	3.11(1.67,5.81)***	1.68(0.90,3.16)
Lowest baseline, highest follow-up (*n* = 114)	0.26(0.13,0.48)***	0.23(0.10,0.56)***	0.27(0.10,0.75)***
Lowest baseline, lowest follow-up (*n* = 290)	1	1	1

Model 1 included adjustment for age, sex and ethnic group. Model 2 included additional adjustment for weight change and the baseline variables of smoking, manual occupation, sedentary behaviour, weight, height, physical activity (quintile and linear term models only), self-rated health, coronary heart disease, diabetes, hypertension, asthma and arthritis.

^a^For physical activity trajectory models, analyses were based on tertile of baseline and follow-up physical activity: low <0.8 h/wk, moderate 7.5–10.8 h/wk and high >10.7 h/wk).

*P < 0.05, **P < 0.01, ***P < 0.001.

### Combined effects of weight change and physical activity change

**Figure 1.**
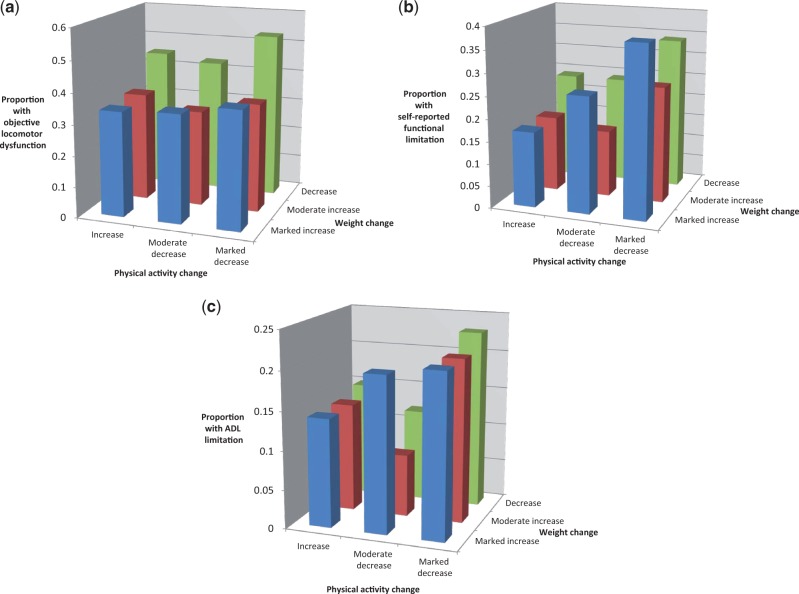
**(a)** The combined effects of weight and physical activity change on objective locomotor dysfunction. (**b**) The combined effects of weight and physical activity change on self-reported functional limitation. (**c**) The combined effects of weight and physical activity change on impairment of activities of daily living.

There was little evidence for an interaction between weight change and physical activity change in models of disability outcomes. However, inspection of plots of the proportion of participants experiencing disability, by weight and physical activity change category, suggested that weight gain in association with increased physical activity appeared to have a less detrimental effect on the likelihood of disability (for all three disability outcomes) than weight gain with reduced or no change in physical activity (Figure 1).

### Sensitivity analyses

Similar patterns for associations between weight and physical activity change and all disability outcomes were seen when analyses were restricted to those without baseline disability, and in younger and older age groups. Including baseline and follow-up weight values in the analyses, instead of weight change, did not change the main results, demonstrating a strong impact of follow-up weight relative to baseline values (results not shown). The relationships between weight change and disability appeared weaker among ‘quitters’ than never smokers (though interpretation was difficult due to small sample size) (results not shown).

## Discussion

We show that increased weight and reduced physical activity over 20 years were separately associated with an elevated likelihood of disability, independent of baseline weight and physical activity, SEP, other health behaviours and chronic disease comorbidities. Weight gain of over 20% and reduction in physical activity of over 7 h per week were associated with around a 2-fold increased odds of physical disability, compared with those whose weight or physical activity remained stable. In addition, it was observed that sustained overweight and obesity during middle and older age had a cumulative relationship with the odds of disability in later life. Thus both weight gain and maintenance of obesity were associated with excess physical disability in older people. Our findings also indicate that the effects of weight gain on disability might be lessened by concurrent increases in physical activity.

This study is unique in its inclusion of a performance-based measure of locomotor dysfunction, directly-measured anthropometry and long follow-up. The majority of other longitudinal studies have been restricted to follow-up periods of less than 5 years;^10^^,^^11^^,^^13^^,^^23^^,^^26–28^ short follow-ups allow for more accurate measurement of weight change, but preclude the observation of the impact of such changes over the long term on important health outcomes.

There are some limitations to consider. Some people may have experienced weight cycles within the two time points 20 years apart and our data prevent us from capturing these changes. In general, however, there is a strong stable trend for weight gain with age.^29^ The observational study design prevents causality assessment, and small numbers in certain weight change categories means that there should be cautious interpretation of these effect estimates. There were losses to follow-up in terms of mortality and non-response. Premature mortality is less of a concern when studying determinants of disability in older age, as by definition individuals need to have survived to older age to be at risk. Thus our findings can only be generalizable to this older age group. Our non-responders were heavier and had adverse risk factor profiles for disability at follow-up (though they did not self-report a greater degree of limitation at baseline). Whereas we cannot be sure of the nature of the association between weight and physical activity change with disability in non-responders, it is unlikely that weight gain, or reduction in physical activity, would be markedly beneficial for disability, nullifying our observed associations. More likely, the reason for non-response could include a greater degree of disability, in part as a consequence of weight gain, which would indicate that we may have underestimated the true effect of these risk factors on disability. Additionally, we performed the main analyses on participants regardless of baseline disability status, as our baseline and follow-up disability measures did not correspond. Although sensitivity analyses on participants with baseline disability demonstrated the same patterns of disability risk, a drawback of our data is the inability to make inferences regarding incident disability. Despite the measurement of anthropometry by trained researchers, errors in measurement were possible and the self-reported behavioural data means that these data may have lacked accuracy. It will be important for future studies to use objectively-measured behaviours, such as actigraphy, to examine the objective impact of these behaviours over time.

Findings have been mixed regarding the impact of directly-measured weight and height on disability. Two studies showed that weight loss predicted an increased risk of incident disability over 5 years,^7^^,^^8^ yet both failed to demonstrate a relationship between weight gain and disability. Another study showed an adverse effect of both weight gain and loss on disability, and the effect of physical strength and clinical disease largely explained the latter.^13^ We observed some increase in disability in the group who went from overweight to healthy, which may perhaps reflect unintentional weight loss due to disease, but the number of people experiencing such weight loss was small and we should interpret these findings with caution. The weak relationships between weight loss and disability in our study could result from the absence of a marked weight loss group, who might have demonstrated the associated elevated risk of disability observed elsewhere. It is possible such individuals may have been lost to follow-up due to ill health. The conflicting findings in the literature are likely to result from variations in weight change thresholds, disability outcomes and follow-up periods and inconsistent adjustment for covariates.

Of interest here is also the independence of the impact of physical activity change on disability from baseline physical activity levels and weight, and weight change. It was surprising to observe the comparable rates of disability among people with high mid-life physical activity reducing to low levels by older age and those with low levels throughout. It is possible that many of those with declining activity experienced ill health following baseline measurement, that caused more sedentary behaviour and disability. This may also explain why the impact of weight gain on disability appeared to be more adverse in the presence of reduced physical activity, and vice versa. Unfortunately our data preclude further exploration of this finding. Our physical activity findings highlight another key public health message: irrespective of body weight, prioritizing exercise promotion throughout the life course has important functional outcomes for older adults. This is supported by data from the NHANES I follow-up study, showing that increased and decreased physical activity were associated with a reduced and an elevated risk of disability, respectively, independent of a range of covariates including baseline physical activity.^15^ Trial data also suggest that increased physical activity reduces disability,^30^ although other studies do not confirm this.^16^

A number of mechanisms through which weight gain influences physical functioning have been proposed, such as increased skeletal stress, loss of muscle mass, atherogenesis and elevated risk of other chronic diseases.^13^^,^^31^ Sarcopenia, the loss of skeletal muscle mass associated with ageing, is thought to be a major contributor to disability in older age,^17^ which may complicate associations between weight gain and disability, since this decline in lean mass may be associated with fat gain but overall weight loss. The relationship between weight gain and disability was shown here to be independent of a range of chronic diseases, including self-reported health, likely capturing unmeasured chronic morbidity. The pathways linking increased activity with reduced disability are similar to those involved in the weight gain-disability relationship, including reduced chronic disease risk, maintenance of muscle mass and strength, improved exercise capacity, flexibility and immune function and increased bone density.^14^^,^^16^^,^^32^^,^^33^

Our findings that weight and behaviour change between mid and late life predict physical disability hold an important public health message. Healthy weight and activity maintenance throughout the life course are key to optimal physical functioning and preservation of independence and quality of life in older people. These findings were robust across sex and ethnic groups. Although the cardiovascular risk associated with obesity may be decreasing, evidence suggests that the burden of disability in obese groups is not showing the same declining trends.^34^ Given the obesity epidemic in all age groups and the growing ageing population globally, understanding the thresholds of weight and activity change that lead to detrimental effects on functioning is paramount for the reduction in disability burden among overweight and obese groups.

## Funding

The follow-up study was supported by a joint programme grant from the Wellcome Trust (082464/Z/07/Z) and British Heart Foundation (BHF) (SP/07/001/23603). The pilot follow-up study was supported by the BHF. The Southall Study was supported by the UK Medical Research Council, the British Diabetic Association (now Diabetes UK), the Wellcome Trust and the BHF. The Brent Study was supported by the UK Medical Research Council. E.D.W. was funded by a Diabetes UK fellowship (09/0003833). The funders played no role in the study design and conduct, in these analyses or in the decision to submit the manuscript for publication.
